# Optimizing an Emergency Medical Dispatch System to Improve Prehospital Diagnosis and Treatment of Acute Coronary Syndrome: Nationwide Retrospective Study in China

**DOI:** 10.2196/36929

**Published:** 2022-11-23

**Authors:** Xuejie Dong, Fang Ding, Shuduo Zhou, Junxiong Ma, Na Li, Mailikezhati Maimaitiming, Yawei Xu, Zhigang Guo, Shaobin Jia, Chunjie Li, Suxin Luo, Huiping Bian, Gesang Luobu, Zuyi Yuan, Hong Shi, Zhi-jie Zheng, Yinzi Jin, Yong Huo

**Affiliations:** 1 Department of Global Health School of Public Health Peking University Beijing China; 2 Institute for Global Health and Development Peking University Beijing China; 3 Emergency Medical Dispatcher Industries Beijing China; 4 Department of Cardiology Shanghai Tenth People’s Hospital Shanghai China; 5 Department of Cardiology Surgery Tianjin Chest Hospital Tianjin China; 6 Department of Cardiology General Hospital of Ningxia Medical University Ningxia China; 7 Department of Cardiology The First Affiliated Hospital of Chongqing Medical University Chongqing China; 8 Department of Cardiology Qinghai Province Cardio-cerebrovascular Disease Specialist Hospital Xining China; 9 Department of Cardiology Tibet Autonomous Region People's Hospital Lasa China; 10 Department of Cardiology The First Affiliated Hospital of Xi’an Jiaotong University Xi'an China; 11 Chinese Medical Association Beijing China; 12 Department of Cardiology Peking University First Hospital Beijing China

**Keywords:** medical priority dispatch system, acute coronary syndrome, prehospital care, emergency medical service, health service, healthcare, health care, coronary, cardiology, cardiovascular

## Abstract

**Background:**

Acute coronary syndrome (ACS) is the most time-sensitive acute cardiac event that requires rapid dispatching and response. The medical priority dispatch system (MPDS), one of the most extensively used types of emergency dispatch systems, is hypothesized to provide better-quality prehospital emergency treatment. However, few studies have revealed the impact of MPDS use on the process of ACS care.

**Objective:**

This study aimed to investigate whether the use of MPDS was associated with higher prehospital diagnosis accuracy and shorter prehospital delay for patients with ACS transferred by an emergency medical service (EMS), using a national database in China.

**Methods:**

This retrospective analysis was based on an integrated database of China’s MPDS and hospital registry. From January 1, 2016, to December 31, 2020, EMS-treated ACS cases were divided into before MPDS and after MPDS groups in accordance with the MPDS launch time at each EMS center. The primary outcomes included diagnosis consistency between hospital admission and discharge, and prehospital delay. Multivariable logistic regression and propensity score–matching analysis were performed to compare outcomes between the 2 groups for total ACS and subtypes.

**Results:**

A total of 9806 ACS cases (3561 before MPDS and 6245 after MPDS) treated by 43 EMS centers were included. The overall diagnosis consistency of the after MPDS group (Cohen κ=0.918, *P*<.001) was higher than that of the before MPDS group (Cohen κ=0.889, *P*<.001). After the use of the MPDS, the call-to-EMS arrival time was shortened in the matched ACS cases (20.0 vs 16.0 min, *P*<.001; adjusted difference: –1.67, 95% CI –2.33 to –1.02; *P*<.001) and in the subtype of ST-elevation myocardial infarction (adjusted difference: –3.81, 95% CI –4.63 to –2.98, *P*<.001), while the EMS arrival-to-door time (20.0 vs 20.0 min, *P*=.31) was not significantly different in all ACS cases and subtypes.

**Conclusions:**

The optimized use of MPDS in China was associated with increased diagnosis consistency and a reduced call-to-EMS arrival time among EMS-treated patients with ACS. An emergency medical dispatch system should be designed specifically to fit into different prehospital modes in the EMS system on a regional basis.

## Introduction

An emergency medical dispatch system is the principal link between the public caller requesting urgent medical care and the emergency medical service (EMS) system, and forms an integral part of EMS practice [1,2]. With proper training, administration, and supervision, an emergency medical dispatcher can accurately query the caller, select an appropriate method of response, provide patient information to responders, and provide appropriate medical direction for patients through the caller. Thus, emergency medical dispatch functions through rapid recognition, rapid dispatching based on priority, and prehospital instructions [3]. Acute coronary syndrome (ACS) is the most time-sensitive acute cardiovascular disease, which requires rapid dispatching and response to dispatching beginning at the time of symptom onset [4]. Timely reperfusion therapy for ACS can be highly effective if following a “chain of survival,” which consists of 3 key components: (1) early symptom recognition and call for EMS, (2) early transportation and evaluation, and (3) early in-hospital treatment. Through appropriate application and reference to a written, medically approved, emergency medical dispatch protocol, an emergency medical dispatch system can lead to a higher diagnosis accuracy and a shorter prehospital delay, which modulates better outcomes for patients with ACS [5-7].

As one of the emergency medical dispatch systems, the medical priority dispatch system (MPDS) has been widely used in more than 50 countries covering more than 3500 EMS centers. MPDS is a scripted protocol designed to direct certified dispatchers to identify the presented symptoms and provide prehospital medical directions based on callers’ responses to scripted questions [8]. MPDS were introduced in China in 2010 and were quickly developed and applied in more than 80 EMS centers after the National Health Commission implemented the Notice on Strengthening the Capacity of Healthcare Delivery for Acute Cardiovascular Diseases in 2015. However, in addition to dispatching and EMS responses, patients with ACS need coordinated care between EMS and hospitals at the regional level, in which EMS providers obtain prehospital electrocardiograms and activate cardiac catheterization laboratories before hospital arrival, bypass the emergency department when appropriate, and provide ongoing quality review and feedback [9]. Therefore, efforts should be focused on information sharing among dispatchers, EMS providers on ambulance, and health care professionals in hospitals. 

In China, the EMS framework was designed specifically to fit into the local health care system. Based on the department in charge of dispatching, prehospital transport, and in-hospital treatment functions, there are at least 4 prehospital EMS system models varying across cities: independent, prehospital, dispatching, and dependent models [10] (Multimedia Appendix 1). The dependent model is the main one encompassing more than 80% of the EMS centers, and the dispatching and independent ones only exist in a few developed cities [11]. The MPDS of China has taken the lead in establishing an information sharing system by linking the EMS and the hospitals to facilitate the coordination of care at the time of entering the EMS system. The MPDS of China is optimized in that it has focused on the establishment of regional systems of ACS care by integrating health care among EMS providers, emergency departments physicians, cardiologists, and catheterization laboratory staff.

A number of studies have verified the accuracy of MPDS dispatch codes in regard to prehospital acuity [12-14]. Prior studies focused on the impact of MPDS use on patients’ outcomes were limited to out-of-hospital cardiac arrest cases [15-17]. However, few studies have revealed the impact of MPDS use on the process of ACS care. Moreover, to our knowledge, no studies have focused on the effectiveness of MPDS use in China and other low- and middle-income countries. To fill the gaps, the objective of this study was to investigate whether the use of the optimized MPDS is associated with higher diagnostic accuracy and a shorter prehospital delay among EMS-treated patients with ACS, using a national database in China. In our patient cohort, ACS was further divided into 3 subtypes: ST-elevation myocardial infarction (STEMI), non–ST-elevation myocardial infarction (NSTEMI), and unstable angina pectoris (UA).

## Methods

### Study Design and Data Source

This retrospective analysis was based on the database of the China MPDS registry and its registered hospitals from January 1, 2016, to December 31, 2020. Data on registered hospitals were extracted from the Chinese Cardiovascular Association Database-Chest Pain Center—a nationwide, web-based, unified database that collects data of patients discharged from the hospital-based chest pain centers [18]. The MPDS registry database collected information of all the EMS users of the MPDS across China, including the name of EMS centers, the date of their official launching of the MPDS, and the code of the administrative region covered by their service.

Based on the date of the official launch of the MPDS at each EMS center, enrolled cases within the EMS service regions were divided into the before MPDS and after MPDS groups. The before MPDS group included cases enrolled in the registered hospitals that had not implemented the MPDS, and the after MPDS group included cases enrolled in the registered hospitals’ chest pain centers that had implemented the MPDS.

### Study Population

**Figure 1:**
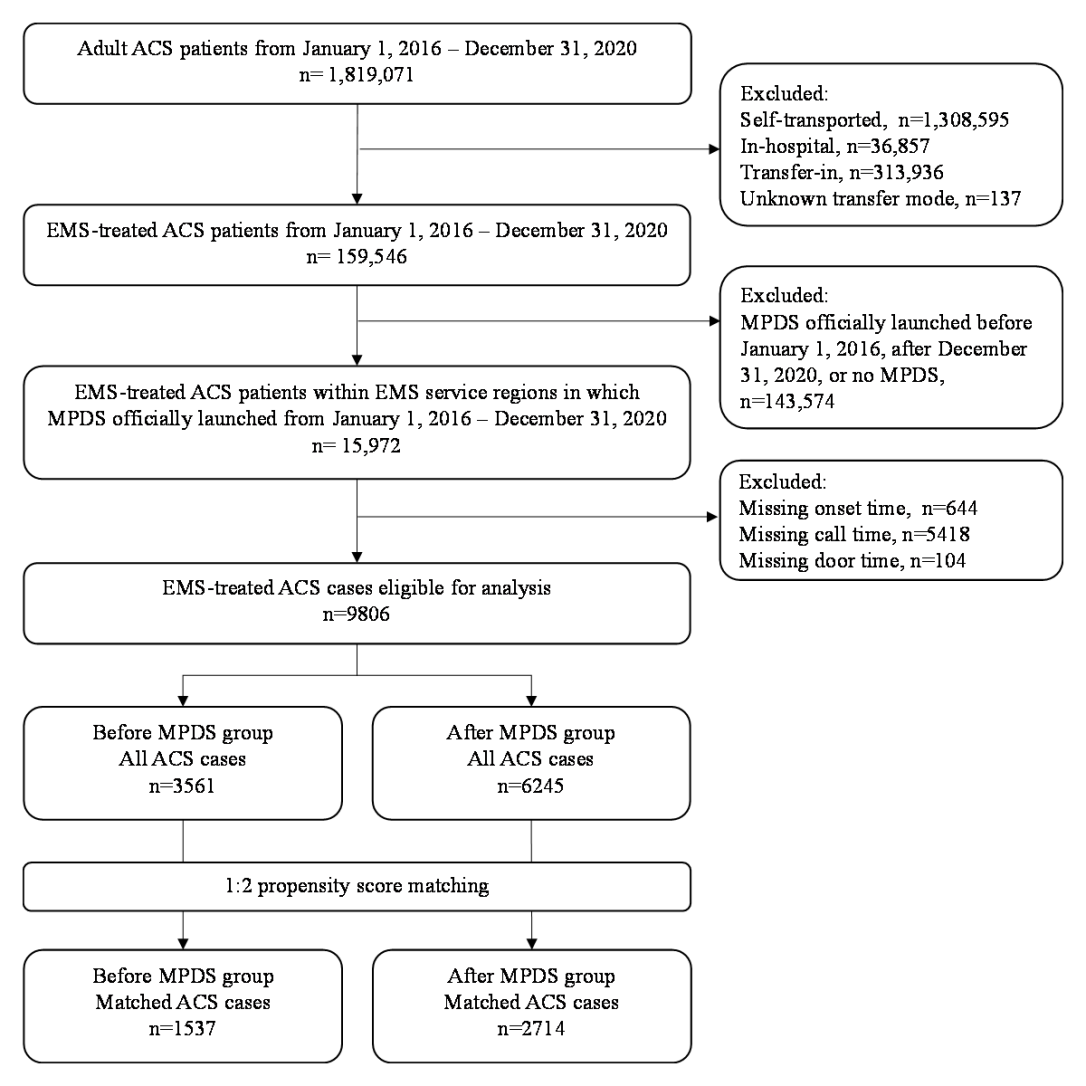
Flow diagram for study recruitment. ACS: acute coronary syndrome; EMS: emergency medical service; MPDS: medical priority dispatch system.

From January 1, 2016, to December 31, 2020, a total of 15,972 patients with a discharge diagnosis of ACS (STEMI, NSTEMI, and UA subtypes) were enrolled in the registered hospitals and treated at a total of 43 EMS centers. A total of 6166 patients were excluded owing to missing data on analyzed indicators including onset time, call time, and door time. Finally, 9806 ACS cases were included in the final analyses and were divided into the before MPDS (n=3561) and after MPDS (n=6245) groups (Figure 1).

### Measures

Primary outcomes included diagnosis consistency and prehospital delay. The diagnosis consistency between diagnosis upon hospital admission (the prehospital diagnosis by the EMS crew) and diagnosis at hospital discharge was indicated using the Cohen κ value. Cohen κ is one of the most common statistics to test interrater reliability and is used to measure the agreement of 2 raters or methods rating on categorical scales [19]. We computed the Cohen κ value to assess the agreement in diagnosing 3 specific subtypes of ACS (STEMI, NSTEMI, and UA) between hospital admission diagnosis and hospital discharge diagnosis.

The prehospital delay was measured by the call-to-EMS arrival time (the time interval from the EMS dispatcher receiving the emergency call from the patient or bystander to ambulance arrival at the scene), the EMS arrival-to-door time (the time interval from EMS arrival at the scene to EMS arrival at the hospital), and total EMS time (the time interval from the EMS dispatcher receiving the emergency call to EMS arrival at the hospital). Covariates for prehospital delay included patients’ demographic characteristics (age and gender), onset environment (city level, call time of the day, and call time of the week), and event characteristics (onset-to-call time, precall chest pain symptoms, type of ACS, and Killip class).

### Data Analysis

We compared the characteristics and outcomes of the study population between the before MPDS and after MPDS groups using a 2-tailed independent samples *t* test and Wilcoxon signed-rank test for continuous variables and the chi-square test for categorical variables. Continuous variables are reported as mean (SD) or median (IQR) values; categorical variables, as n (%) values. To examine the impact of the optimized MPDS on prehospital delay, we used 2 models including propensity score–matching analysis and multivariable logistic regression analysis. Both models were adjusted for precall covariates including patients’ demographic characteristics (age and gender), onset environment (city level, call time of the day, and call time of the week), and event characteristics (onset-to-call time, precall chest pain symptoms, type of ACS, and Killip class), with *P*<.05 considered the threshold for statistical significance. In the propensity score–matching analyses, 1:2 matching was performed without replacement for each patient, using a nearest-neighbor matching algorithm with a caliper width of 0.02. Matched patients were compared to assess balance in covariates (ie, standardized differences for each covariate were <10%). In the multivariable logistic regression analysis, adjusted differences with 95% CIs are presented. All statistical analyses were performed using R (version 4.0.4; The R Foundation).

### Ethical Considerations

This study was approved by the institutional review board of Peking University (IRB00001052-21020). Informed consent was obtained from all participants prior to questionnaire administration.

## Results

### Patient Characteristics

Compared to the before MPDS group (n=3561), the after MPDS group (N=6245) comprised younger patients (mean 65.6, SD 12.9 vs mean 66.2, SD 13.4 years, respectively, *P*=.03), had a higher proportion of cases from provincial capital cities (41.4% vs 32.4%, *P*<.001), and had a higher proportion of patients with STEMI (62.9% vs 57.8%, *P*<.001) and Killip class I myocardial infarction (77.8% vs 72.8%, *P*<.001). After propensity score–matching, 2715 patients in the before MPDS group and 5429 patients in the after MPDS group were matched (Table 1).

**Table 1:** Characteristics and outcomes of acute coronary syndrome cases, before and after dispatch with the optimized medical priority dispatch system (MPDS), total cases (N=9806), and propensity score–matched cases.

Characteristics and Outcomes		Total cases			Propensity score–matched cases^a^ (n=8144)			Standardized mean difference
		Before MPDS (n=3561)	After MPDS (n=6245)	*P* value	Before MPDS (n=2715)	After MPDS (n=5429)	*P* value	

Age (years), mean (SD)		66.2 (13.4)	65.6 (12.9)	.03	62.6 (13.3)	65.2 (12.9)	.25	0.02
Male, n (%)		2588 (72.7)	4414 (70.7)	.04	2008 (74.0)	3888 (71.6)	.03	0.05
Living in a provincial capital city, n (%)		1168 (32.4)	2587 (41.4)	<.001	1027 (37.8)	2251 (41.5)	.002	0.20
**Call time of the day, n (%)**				.10			.07	0.06
	12-5:59 AM	651 (18.3)	1252 (20.0)		491 (18.1)	1089 (20.1)		
	6-11:59 AM	1154 (32.4)	2030 (32.5)		860 (31.7)	1765 (32.5)		
	12-5:59 PM	875 (24.6)	1518 (24.3)		690 (25.4)	1324 (24.4)		
	6-11:59 PM	881 (24.7)	1445 (23.1)		674 (24.8)	1251 (23.0)		
Call on weekday, n (%)		2516 (70.7)	4505 (72.1)	.12	1914 (70.5)	3925 (72.3)	.09	0.04
**Precall chest pain, n (%)^b^**				.004			.21	0.04
	Persistent chest pain	2233 (69.1)	4221 (72.1)		1954 (72.0)	3960 (72.9)		
	Intermittent chest pain	837 (25.9)	1336 (22.8)		644 (23.7)	1206 (22.2)		
	Eased chest pain	163 (5.0)	299 (5.1)		117 (4.3)	263 (4.8)		
**Type of acute coronary syndrome, n (%)**				<.001			.21	0.06
	ST-elevation myocardial infarction	2057 (57.8)	3928 (62.9)		1703 (62.7)	3508 (64.6)		
	Non–ST-elevation myocardial infarction	627 (17.6)	1084 (17.4)		554 (20.4)	971 (17.9)		
	Unstable angina pectoris	877 (24.6)	1233 (19.7)		458 (16.9)	950 (17.5)		
**Killip class, n (%)^b^**				<.001			.05	0.07
	I	2333 (72.8)	4495 (77.8)		2073 (76.4)	4269 (78.6)		
	II-III	625 (19.5)	876 (15.2)		440 (16.2)	814 (15.0)		
	IV	245 (7.6)	408 (7.1)		202 (7.4)	346 (6.4)		
Onset-to-call time (minutes), median (IQR)		54.0 (18.0-124.0)	56.0 (20.0-124.0)	.48	56.0 (20.0-128.0)	56.0 (20.0-124.0)	.87	0.03
Call-to-EMS^c^ arrival time (min), median (IQR)		18.0 (12.0-30.0)	16.0 (10.0-26.0)	<.001	20.0 (12.0-30.0)	16.0 (10.0-26.0)	<.001	N/A^d^
EMS arrival-to-door time (minutes), median (IQR)		20.0 (12.0-30.0)	20.0 (12.0-28.0)	<.001	20.0 (12.0-30.0)	20.0 (12.0-30.0)	.31	N/A
Total EMS time (call-to-door; minutes), median (IQR)		40.0 (28.0-56.0)	38.0 (28.0-52.0)	<.001	40.0 (29.0-58.0)	38.0 (28.0-52.0)	<.001	N/A

^a^Propensity score matched for age, gender, city level, call time of the day, call on weekday, precall chest pain symptoms, type of acute coronary syndrome, Killip class, and onset-to-call time.

^b^Missing cases were excluded when comparing the precall chest pain symptoms and Killip class between the 2 groups.

^c^EMS: emergency medical service.

^d^N/A: not applicable.

### Diagnosis Consistency

**Figure 2:**
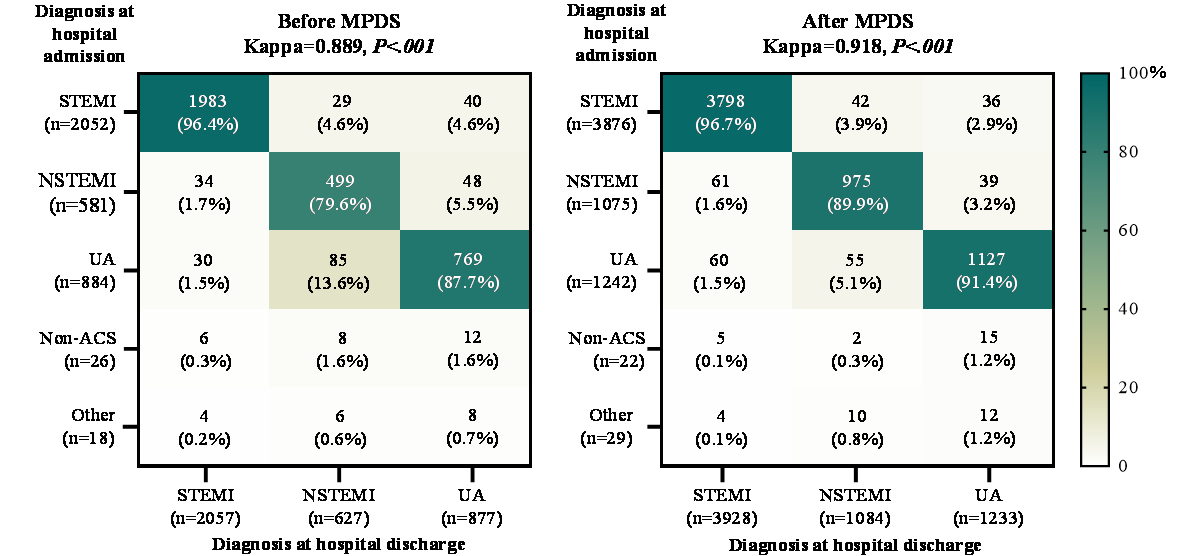
Diagnosis consistency between hospital admission and discharge of EMS-treated patients with ACS before and after dispatch with the optimized MPDS. Data are reported as the proportion of each diagnosis upon admission among the cases with certain diagnosis at discharge. Cohen κ values were computed in 3 certain types of ACS (STEMI, NSTEMI, UA) diagnosis between admission and discharge. Non-ACS cases include pulmonary embolism, aortic dissection, and non-cardiogenic chest pain. Other cases include non–chest pain and unknown diagnosis. ACS: acute coronary syndrome; MPDS: medical priority dispatch system; STEMI: ST-elevation myocardial infarction; NSTEMI: non–ST-elevation myocardial infarction; UA: unstable angina pectoris.

The Cohen κ of all ACS subtypes was higher in the after MPDS group (0.918, *P*<.001) than in the before MPDS group (0.889, *P*<.001). Specifically, diagnosis consistency of NSTEMI (79.6% vs 89.9%, *P*<.001) and UA (87.7% vs 91.4%, *P*=.001) were remarkably improved after the use of the optimized MPDS, while that of STEMI (96.4% vs 96.7%, *P*>.99) was not significantly changed (Figure 2). Moreover, 44 of 3561 (1.2%) of patients in the before MPDS group and 51 of 6245 (0.8%) patients in the after MPDS group with a discharge diagnosis of ACS chest pain were treated for non-ACS chest pain or other diseases upon admission.

### Prehospital Delay

In the propensity score–matched population, call-to-EMS arrival time (20.0 vs 16.0 minutes, *P*<.001) and the total EMS time (40.0 vs 38.0 minutes, *P*<.001) were significantly shorter after the use of MPDS, while the EMS arrival-to-door time (20.0 vs 20.0 minutes, *P*=.31) between the before and after MPDS groups were not significantly different (Table 1).

**Figure 3:**
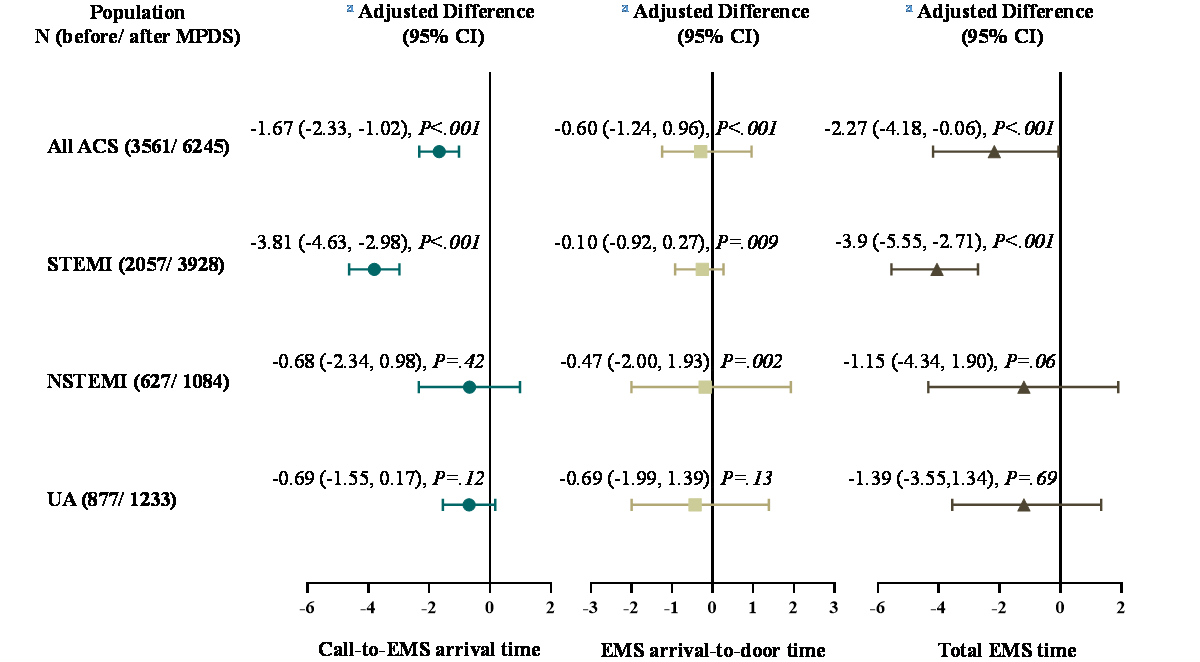
Multivariate analysis of outcomes using a generalized linear model in all ACS cases and for each subgroup with STEMI, NSTEMI, and UA. The model was adjusted for age, gender, city level, call time of the day, and calling on a weekday. Call-to-EMS arrival time was defined as the time interval from EMS dispatcher receiving the emergency call to ambulance arrival at the scene; the EMS arrival-to-door time was defined as the time interval from EMS arrival at the scene to EMS arrival at the hospital; total EMS time was defined as the time interval from the EMS dispatcher receiving the emergency call to EMS arrival at the hospital. ACS: acute coronary syndrome; EMS: emergency medical service; MPDS: medical priority dispatch system; NSTEMI: non–ST-elevation myocardial infarction; STEMI: ST-elevation myocardial infarction; UA: unstable angina pectoris.

Patients in the after MPDS group had a significantly shorter call-to-EMS arrival time than those in the before MPDS group in all ACS cases (adjusted difference –1.67, 95% CI –2.33 to –1.01, *P*<.001), and those of the STEMI subtype (adjusted difference –3.81, 95% CI –4.63 to –2.98, *P*<.001). There were no significant differences between the 2 groups in EMS arrival-to-door time in all ACS cases or subtypes (Figure 3).

## Discussion

### Principal Findings

In this retrospective study of EMS-treated patients based on a national database in China, we found that the use of the optimized MPDS was associated with a higher consistency between diagnosis at hospital admission and discharge and a shorter call-to-EMS arrival time; however, there were no significant differences in the EMS arrival-to-door time among patients with ACS. Our findings are consistent with those of prior studies in which the use of MPDS has been proven to be associated with high dispatching accuracy [12,13] and improved dispatch efficacy [14], which could potentially prove the general assumption that MPDS could provide higher diagnosis accuracy and lesser prehospital delay, thereby potentially resulting in better survival outcomes for ACS.

The first assumption was that the optimized MPDS could help rapidly identify and diagnose diseases, which theoretically led to a higher diagnostic accuracy of EMS. In this study, we observed an increase in overall diagnosis consistency between hospital admission and discharge after the use of the optimized MPDS, which was similar to the diagnostic accuracy of ACS in China that reported elsewhere [20,21]. This suggests that although the optimized MPDS might not provide a definite diagnosis for each case, it has the potential to allocate patients to the right priority levels in accordance with their symptom presentation. In fact, the MPDS was purposefully designed to be highly sensitive and to avoid undertriage by creating overtriage so as to ensure patient care and safety at the first place [12,22,23]. We also observed that a lower proportion of patients with ACS were treated for other diseases upon admission, which might lead to reduced wastage of resources and risk for personnel [24]. Nevertheless, our findings once again revealed the complexity of the diagnosis of ACS.

The second assumption was that the optimized MPDS could reduce prehospital delay through timely dispatch and appropriate EMS responses. In fact, the use of the optimized MPDS reduced the transportation delay in the call-to-EMS arrival time; however, it did not translate to a shorter EMS arrival-to-door time. On the one hand, although the MPDS of China has taken efforts to establishing the information sharing system to integrate health care between the EMS and the hospital-based chest pain centers, it was still only involved in the process from call receiving to EMS arrival at the scene. On the other hand, the reduced call-to-EMS arrival time indicated the adaptability of the optimized MPDS in China’s EMS system at the local level. As indicated, the varied EMS systems in China could be classified into 4 main models. In spite of different characteristics, all 4 models could present prehospital delay. The independent model and prehospital model tend to have longer dispatching and ambulance returning times, especially within broad regions with limited health resources. In these cities, the optimized MPDS’s priorities could help dispatchers mobilize health resources, which may avoid unnecessary wastage of health resources, thus shortening the time of dispatching and arriving at the scene. For the dispatching model and dependent model, the response speed of hospitals may be worse than expected owing to limited authority of the EMS, leading to low response to dispatching. The optimized MPDS follows standardized procedures and records detailed registration of every emergency call and would empower the EMS with greater authority, which may improve the responsiveness of hospitals to dispatching, thus reducing the call-to-EMS arrival time. Therefore, to further improve the impact of the optimized MPDS, the optimized MPDS should be designed specifically to fit into different prehospital models of the EMS system on a regional basis.

The third assumption was that with a higher diagnostic accuracy and a shorter prehospital delay, the optimized MPDS could result in better survival outcomes for ACS. Though the outcome data could not be obtained and analyzed in this study, the onset-to-call time was still near 1 hour; thus, the optimized MPDS could hardly predict improved in-hospital mortality. For time-concerning emergencies such as ACS, the first link of the chain of survival would always be early symptom recognition and seeking for EMS by the public [25-27]. Any subsequent treatment will not be effective without timely activation of this first link. Given the fact that 1-year mortality for patients with ACS would increase by 7.5% with every additional 30 minutes of prehospital delay [4], this large period between symptom onset to call would always limit what the optimized MPDS can do. Therefore, what should be designed in a dispatching system and whether its implementation can result in satisfactory effects not only depend on the EMS but also require the joint efforts of the public, EMS, and hospitals.

### Limitations

This study had some limitations. First, this was a retrospective study, which increased the risk of residual confounding. Although we eliminated imbalance between the groups through propensity score–matching analysis, unmeasured confounding factors may have influenced the outcomes. Second, our study population comprised EMS-treated patients enrolled at chest pain centers, and all patients were at least alive at the time of admission, which might limit the generalizability of our findings. However, our comparison between propensity score–matched groups was able to eliminate this bias. Third, we failed to classify our included EMS systems into specific types of EMS models because of a lack of an official classification, which may limit the certainty of our findings. Fourth, owing to limited variables in the database, we could not obtain the survival outcome; we failed to determine the call processing time, the ambulance dispatch time, or EMS on-scene time, which would affect the prehospital delay and could be impacted by the MPDS; for the measures of diagnosis accuracy of the optimized MPDS, we could only compute the Cohen κ using the disease diagnosis rather than priority levels, while the sensitivity and specificity for discriminative, positive, and negative predictive values could not be obtained.

### Conclusions

The use of the optimized MPDS in China was associated with a higher diagnosis consistency and a shorter call-to-EMS arrival time; however, no potentially improved EMS-to-door time among EMS-treated patients with ACS. These benefits can be realized by the emergency medical dispatch system when coordinated care between the EMS and hospitals was delivered on the regional level.

## Appendix

Multimedia Appendix 1 Prehospital modes of China’s EMS system.

## Abbreviations

ACS: acute coronary syndrome
EMS: emergency medical service
MPDS: medical priority dispatch system
NSTEMI: non–ST-elevation myocardial infarction
STEMI: ST-elevation myocardial infarction
UA: unstable angina pectoris
