# Supplementary material for: Optimizing an Emergency Medical Dispatch System to Improve Prehospital Diagnosis and Treatment of Acute Coronary Syndrome: Nationwide Retrospective Study in China
Source: J Med Internet Res. 2022 Nov 23;24(11):e36929. doi: 10.2196/36929 (PMC9730207; doi:10.2196/36929)
Supplement: Multimedia Appendix 1 [file jmir_v24i11e36929_app1.docx]

**Appendix Table 1**. Prehospital modes of China’s EMS system

| **Prehospital modes** | **Dispatching** | **Pre-hospital transport** | **In-hospital treatment** |
| --- | --- | --- | --- |
| Independent model | EMS | EMS | EMS |
| Prehospital model | EMS | EMS | Hospitals |
| Dispatching model | EMS | Hospitals | Hospitals |
| Dependent model  (EMS belongs to one hospital) | Hospitals | Hospitals | Hospitals |

EMS, emergency medical service.
